# Supplementary material for: Diverse Electronic and Magnetic Properties of Chlorination-Related Graphene Nanoribbons
Source: Sci Rep. 2018 Dec 14;8:17859. doi: 10.1038/s41598-018-35627-6 (PMC6294761; doi:10.1038/s41598-018-35627-6)
Supplement: Supplementary file 1 — Supplementary information [file 41598_2018_35627_MOESM1_ESM.pdf]

Diverse Electronic and Magnetic Properties of Chlorination-Related Graphene Nanoribbons

Duy Khanh Nguyen[1], Ngoc Thanh Thuy Tran[2], Thanh Tien Nguyen[3], and Ming-Fa Lin[4,*]

[1]Department of Physics, National Cheng Kung University, Tainan 701, Taiwan
[2]Hierarchical Green-Energy Materials (Hi-GEM) Research Center, National Cheng Kung University, Tainan 701, Taiwan

[3]Department of Physics, College of Natural Sciences, Can Tho University, 3-2 Road, Can Tho, Viet Nam

[4]Hi-GEM/Quantum Topology Research Center, National Cheng Kung University, Tainan 701, Taiwan
[*]mflin@mail.ncku.edu.tw

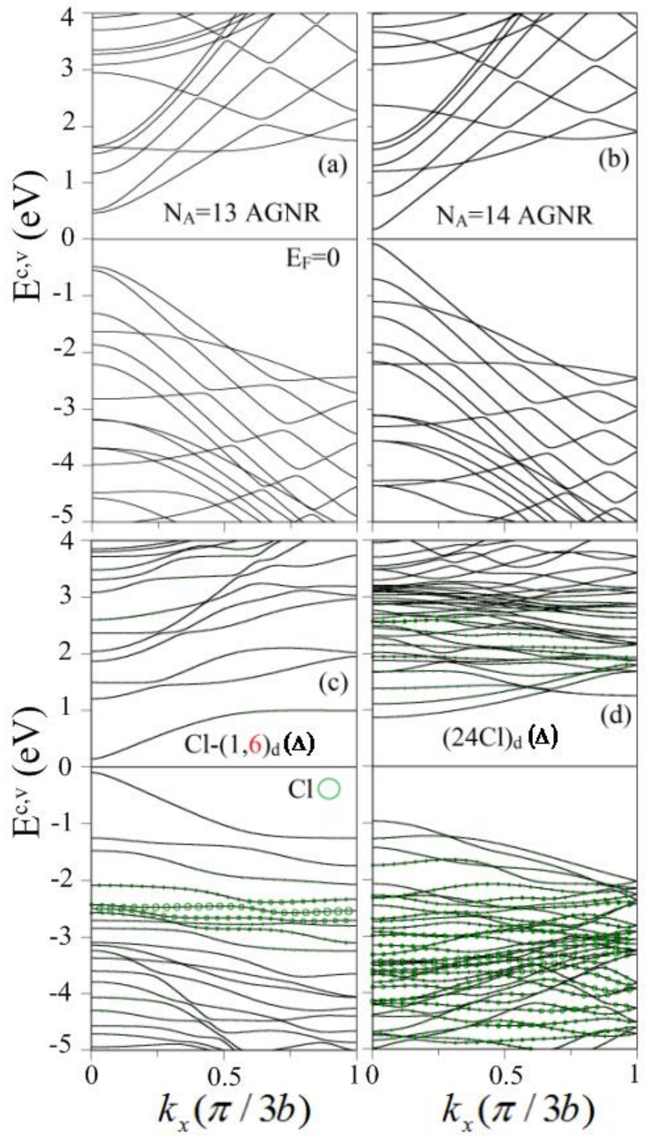

Supplementary Figure. S1. Band structures of (a) N_A=13 AGNR, (b) N_A=14 AGNR, (c) Cl-(1,6)$_d$- and (d) (24Cl)$_d$-adsorbed N_A=12 AGNR. Δ represents the short Cl-C distance.

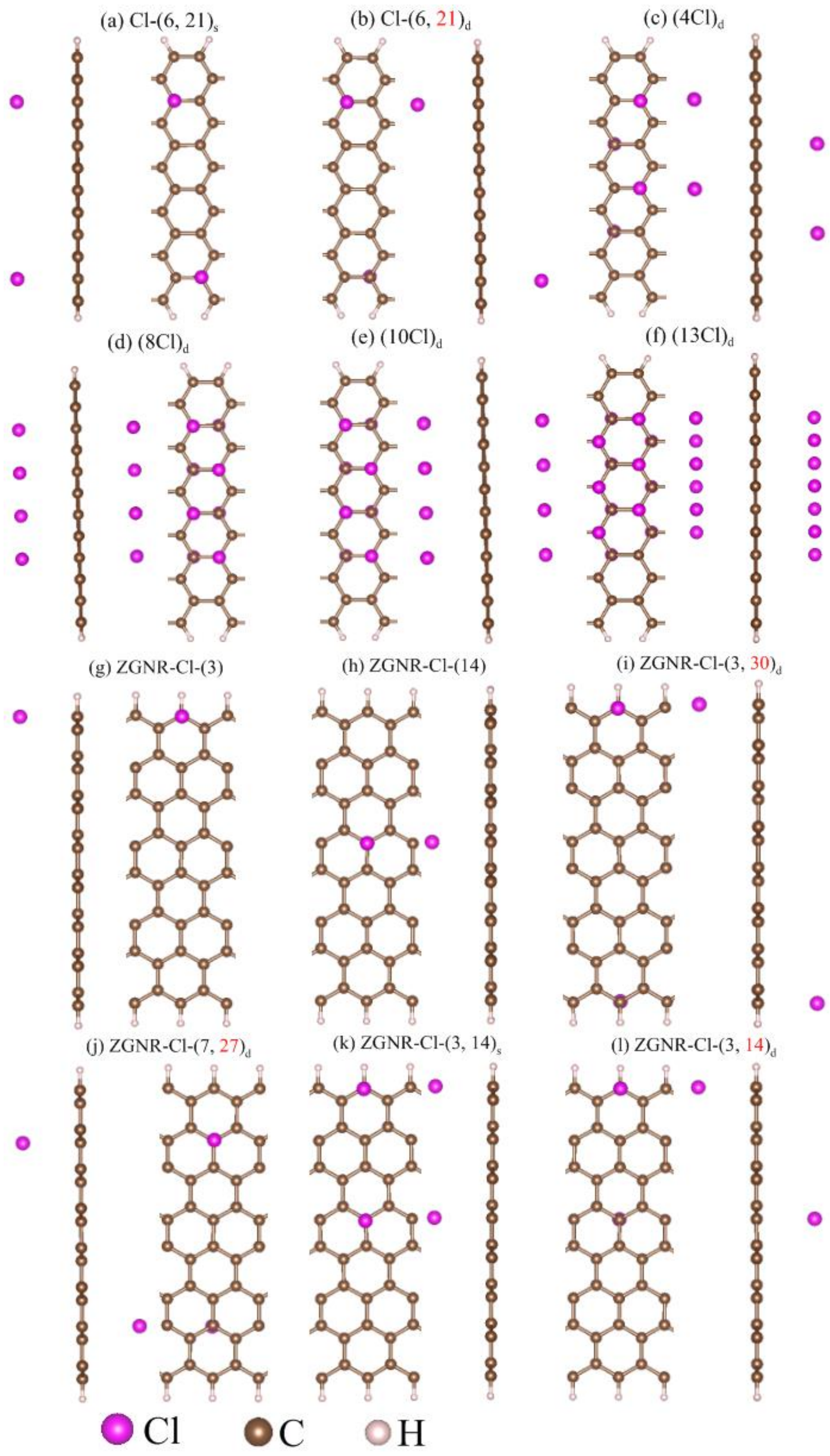

Supplementary Figure. S2. The top- and side-view geometric structures of halogen adatoms-adsorbed GNRs.
